# Retraction: MicroRNA-564 is downregulated in glioblastoma and inhibited proliferation and invasion of glioblastoma cells by targeting TGF-β1

**DOI:** 10.18632/oncotarget.28748

**Published:** 2025-06-17

**Authors:** Chunming Jiang, Fang Shen, Jianmin Du, Zhaoyang Hu, Xiaoli Li, Jin Su, Xiaohua Wang, Xianmei Huang

**Affiliations:** ^1^Department of Pediatrics, Affiliated Hangzhou Hospital of Nanjing Medical University, Hangzhou First People’s Hospital, Hangzhou, Zhejiang 310003, P.R. China; ^2^Department of Psychiatry, Tongde Hospital of Zhejiang Province, Hangzhou, Zhejiang 310012, P.R. China; ^3^Translational Medicine Office of Hangzhou Cancer Institute, Affiliated Hangzhou Hospital of Nanjing Medical University, Hangzhou First People’s Hospital, Hangzhou, Zhejiang 310003, P.R. China; ^4^Department of Pediatrics, The First Affiliated Hospital of Nanjing Medical University, Nanjing, Jiangsu 210029, P.R. China; ^5^Department of Pediatrics, Nanjing First Hospital, Nanjing Medical University, Nanjing, Jiangsu 210029, P.R. China; ^*^Chunming Jiang and Fang Shen are co-first authors

**This article has been retracted:** Oncotarget has concluded its investigation of this paper. Multiple internal and external duplications were revealed. We found that Figures 2C and 5B contain internal image duplications. Furthermore, significant similarities were identified between this article and several publications from other research groups with differing scientific contexts [1–9], most of which have already been retracted. Thus the invasion analysis image in Figure 5B was also found in Figure 3B of [1], Figure 3C of [2], and Figure 2C of [3] (all published prior), and Figure 3G of [4] (published concurrently). Additionally, the Western blot for TGFb in Figure 3D is a duplicate of the Western blot for TIAM1 in Figure 4D of the 2015 publication [5], which also shared the GAPDH blot in Figure 5B with Figure 4A of Oncotarget paper under discussion. This same Western blot for GAPDH in Figure 4A was also found in multiple other publications - in Figures 4A of [6], 7E of [7], 5B of [8] and 4D of [9]. Notably, 2015 paper [6] also shared the GAPDH image in Figure 3D and the structure of this paper is identical to the paper under discussion.

The corresponding author, when reached for comment, explained that a portion of the experiments had been outsourced to a third-party company to help finalize the project, and the company reused the images in other studies. The authors acknowledged that this outsourcing was not disclosed in the paper, took full responsibility for these errors, and apologized to the scientific community.

Given the number and significance of the errors identified in the published figures, the authors and the editors have lost confidence in the presented data. Consequently, the editorial decision to retract this paper was made, and all authors have agreed to this decision.

Original article: Oncotarget. 2016; 7:56200–56208. 56200-56208. https://doi.org/10.18632/oncotarget.8987
